# Dr. Regnault, on the Lichen Islandicus

**Published:** 1804-11-01

**Authors:** J. B. Regnault


### *To the Editors of the Medical and Physical Journal.*

GENTLEMEN,

HAVING been requested, both publicly and privately, to give my opinion of the preparation of the *Lichen Islandicus*, recommended by Mr. Reece in his pamphlet on that subject; and believing that a valuable article of the *Materia Medica* may become inert by improper administration, I have complied with the request.

Mr. Reece condescends to agree with all the celebrated physicians of the North, whose authorities I had quoted respecting the virtues of the Iceland moss, in pulmonary consumption; he also agrees with me, that it should be copiously administered; but he condemns at the same time the mode prescribed for employing it, and prefers his own powder or *farina*. This preference is the point which I am now called upon to consider.

He says, p. 3, of his pamphlet, "The bitter portion of this herb (which must be considered the principal agent in the relief of the phthisical symptoms) is readily imparted to boiling water by infusion; but by the long boiling necessary to extract its mucilage, this quality is nearly destroyed."

This assertion is the very reverse of the truth in every respect; the great virtues of the lichen are contained in a mucilage of a peculiar nature, which is rendered the more effectual by its combination with a bitter principle. I have pointed out, in pages 20, 21 and 22 of my work, the difference between this mucilage and all others hitherto known. This bitter principle does not evaporate by boiling, as Mr. R. erroneously supposes; a continued and uniform boiling is, on the contrary, necessary to extract the virtues of the plant. Cramer, Reiske, Ebeling, Schoneyder, who have all ably written on this vegetable substance, of which they have given different analyses, are of the same opinion. I shall say nothing of Mr. Reece's objections objections to all the preparations of the plant: judgment alone, without any practical knowledge, is sufficient to refute them.

"This preparation of *farina,* (says Mr. R.) is free from the cortical and fibrous part of the herb. It possesses, *in perfection,* both the medicinal and dietetic properties."

Where does Mr. Reece find that the cortical and fibrous parts have nothing to do with the virtues of the herb? Upon what authority does he advance an assertion so contrary to all the facts hitherto known? After assuring us, p. 7, that his preparation possesses, *in perfection,* both the medicinal and dietetic properties, he tells, p. 9, that, "If the phthisical symptoms should indicate the use of the bitter quality of the lichen, *a greater proportion* than that contained in the farina, a concentrated infusion may be made by infusing three ounces of the plant, &c."

We must admit that Mr. R. might have been more consistent, had his partiality for his *farina* allowed it.

Mr. R. is not less apprehensive for the loss of the virtues of the lichen by ebullition, when given as medicine, than when administered as food; for which reason he carefully *avoids* much boiling. But he should know, that the constituent principles of the plant are extremely compact, and so strongly united together, that a considerable degree of boiling is necessary to render it susceptible of being decomposed in our system, and the nutritious part extracted from it. Mr. R. like many others, does not sufficiently discriminate between what is digested and what passes through the body; many vegetable substances are dissolved and absorbed by the whole absorbent system of the intestines, which is called the solution of the aliment, but not its true digestion. An aliment is digested only in as much as it is decomposed, transmuted, and its dissociated elements attracted, each by affinity, to repair the different solid and fluid systems. When, therefore, a vegetable aliment is introduced into the animal economy, without being decomposed, reduced to its elements, and appropriated or assimilated, it is only dissolved, and retains a part of its basis, which is a substance heterogeneous to the animal frame, and incapable of supplying the blood with the necessary principles.

Mr. R. will possibly refer us to the Icelanders, who habitually use the farina of lichen; but the ordinary food of that people is composed of gross aliments, which the inhabitants of our towns could not digest like the Icelander, accustomed to hard labour, and to live in the open and keen air, air, which furnish the blood with the necessary elements that raise the digestive faculties to their greatest height. But how could the digestive juices of our citizens, particularly of those who are weakened by disease, decompose this farinaceous substance, the extremely compact elements of which can only be disunited and rendered capable of assimilation with our fluids, by a preparatory decomposition? Besides, in the different modes of preparing this farina, the Icelanders submit it to a much longer boiling than Mr. Reece.

The above considerations have induced me to combine the Iceland moss with other substances, to be given as food; and with this view I make it undergo a preparatory elaboration, to render it more capable of assimilation with the different systems of the human economy.

I presume what I have said will be sufficient to demonstrate, how much Mr. Reece has been mistaken respecting the use of this herb, both as a medicament and as an aliment.

I now conclude this letter, which I fear is already too long, by referring your readers to the Medical and Physical Journal, for January, 1804, where other mistakes of Mr. R. are noticed, and of which I have made no mention above. I am, &c.

*Oct.* 14, 1804. J. B. REGNAULT, M.D.

